# Characterization of Antibacterial Activities of Eastern Subterranean Termite, *Reticulitermes flavipes*, against Human Pathogens

**DOI:** 10.1371/journal.pone.0162249

**Published:** 2016-09-09

**Authors:** Yuan Zeng, Xing Ping Hu, Sang-Jin Suh

**Affiliations:** 1 Department of Entomology and Plant Pathology, Auburn University, Auburn, AL, United States of America; 2 Department of Biological Sciences, Auburn University, Auburn University, Auburn, AL, United States of America; Institute of Plant Physiology and Ecology Shanghai Institutes for Biological Sciences, CHINA

## Abstract

The emergence and dissemination of multidrug resistant bacterial pathogens necessitate research to find new antimicrobials against these organisms. We investigated antimicrobial production by eastern subterranean termites, *Reticulitermes flavipes*, against a panel of bacteria including three multidrug resistant (MDR) and four non-MDR human pathogens. We determined that the crude extract of naïve termites had a broad-spectrum activity against the non-MDR bacteria but it was ineffective against the three MDR pathogens *Pseudomonas aeruginosa*, methicillin-resistant *Staphylococcus aureus* (MRSA), and *Acinetobacter baumannii*. Heat or trypsin treatment resulted in a complete loss of activity suggesting that antibacterial activity was proteinaceous in nature. The antimicrobial activity changed dramatically when the termites were fed with either heat-killed *P*. *aeruginosa* or MRSA. Heat-killed *P*. *aeruginosa* induced activity against *P*. *aeruginosa* and MRSA while maintaining or slightly increasing activity against non-MDR bacteria. Heat-killed MRSA induced activity specifically against MRSA, altered the activity against two other Gram-positive bacteria, and inhibited activity against three Gram-negative bacteria. Neither the naïve termites nor the termites challenged with heat-killed pathogens produced antibacterial activity against *A*. *baumannii*. Further investigation demonstrated that hemolymph, not the hindgut, was the primary source of antibiotic activity. This suggests that the termite produces these antibacterial activities and not the hindgut microbiota. Two-dimensional gel electrophoretic analyses of 493 hemolymph protein spots indicated that a total of 38 and 65 proteins were differentially expressed at least 2.5-fold upon being fed with *P*. *aeruginosa* and MRSA, respectively. Our results provide the first evidence of constitutive and inducible activities produced by *R*. *flavipes* against human bacterial pathogens.

## Introduction

In recent years, insects have been recognized for having potent immune defenses that produce constitutive and inducible antimicrobial compounds to combat various pathogens [1]. Thus, they have been targeted as a potential source of antimicrobial compounds [2, 3]. Insects possess complex immune responses that act synergistically to provide protection against microbial infections [4]. When pathogens break through morphological barriers, insects evoke innate immune responses comprised of cellular and humoral reactions. Cellular reactions are hemocyte-mediated and include phagocytosis and encapsulation, while humoral reactions involve the production of antimicrobial proteins and activation of enzymatic cascades [5]. Over the last few decades, more than 150 insect antimicrobial peptides/proteins (AMPs) have been identified from naïve, microbe-challenged, or injured insects [6]. Reported insect AMPs include lysozymes, cecropins, attacins, defensins, and proline rich peptides [6, 7].

Recently, several constitutive antimicrobial proteins and peptides have been identified from three termite families: Termopsidae [8], Rhinotermitidae [9–12], and Termitidae [13–16]. The majority of these molecules have antifungal activities and only a few, including termicin, defensin-like peptides, spinigerin, and lysozymes, have weak antibacterial activities [13, 16]. Hussain et al. [17] reported induction of antibacterial activity from the whole body homogenates of *Coptotermes formosanus* Shiraki upon exposure with various bacteria, including a human pathogen *Staphylococcus aureus*. However, exposure to different bacteria did not stimulate activity against the inducing organisms except for *Bacillus thuringiensis*.

Subterranean termites (Blattodea: Isoptera: Rhinotermitidae), especially the *Reticulitermes* species, are widely distributed in the United States. These termites have developed disease resistance mechanisms that facilitated their survival and propagation as they nest and forage in soil [18]. Termite-produced AMPs, termicin (initially isolated from a fungus-growing termite) and tGNBPs (termite gram-negative binding proteins), have been described in the eastern subterranean termite *R*. *flavipes* and the dark southern subterranean termite *R*. *virginicus* [9, 10, 13]. GNBP2 has β-1, 3-glucanase activity in termites and contributes to external antifungal defense [13]. We previously reported the discovery of constitutive antibacterial activity from the cell-free extract (CFE) of *R*. *flavipes* against a common Gram-positive soil-borne entomopathogenic bacterium, *B*. *subtilis* [12]. In this study, we determined the presence, characteristics, and levels of constitutive and inducible antibacterial activities in *R*. *flavipes* against a panel of human bacterial pathogens including three common multidrug resistant nosocomial pathogens and five non-MDR pathogens.

## Materials and Methods

### Termite maintenance and induction of antimicrobial activity

*R*. *flavipes* were collected on the Auburn University campus as previously described [12, 19] and workers were maintained in Urban Entomology Laboratory at 25 ± 2°C for at least 20 days before being subjected to experiments. To examine the potentially inducible antibacterial activity, two heat-killed MDR pathogens, Gram-negative *P*. *aeruginosa* and Gram-positive MRSA, were selected to stimulate the immune response. Bacteria were grown in Lysogeny Broth (LB) [20] at 37°C with aeration overnight, subcultured, and grown in fresh LB to early-mid log-phase (OD_600_ = 0.3 ± 0.05). Twenty-four ml of heat-killed bacterial suspension was obtained as follows: cells from 48 ml of culture was harvested via centrifugation at 13,200 rpm for 5 min, washed twice with 48 ml of milli-Q (MQ) water, resuspended in 24 ml of MQ water (~6 x 10^8^ cells/ml), and heat-killed at 100°C for 10 min. *R*. *flavipes* workers were surface sterilized with 70% ethanol to eliminate surface microbes immediately before being subjected to testing. To immunize the workers, a group of 3 g of surface-sterilized termites (8 groups per treatment) was introduced into Petri plates (15 cm × 2.5 cm) provisioned with sterile filter papers (12.5 cm in diameter, Whatman #1; 1 filter paper per Petri dish) moistened with 3 ml of heat-killed *P*. *aeruginosa* or MRSA suspension, respectively. Sterile filter papers were moisturized with the same amount of MQ water and were used as negative controls. The termites were allowed to feed in Petri plates for 24 hours and then harvested for analysis.

### Preparation of whole body and size-fractionated CFE

Surface sterilized naïve and heat-killed pathogen challenged termite workers (24 g each) were suspended, separately, in 120 ml of 20 mM Tris-HCl, 20 mM NaCl (pH = 7.5) buffer and homogenized as previously described [12]. The crude CFE was quickly frozen in liquid nitrogen and lyophilized (Heto Lyolab 3000, Thermo Fisher Scientific, Pittsburgh, PA) at -57°C overnight before being dissolved in MQ water to achieve the final concentration of approximately 20 mg/ml protein concentration as determined by the Bradford assay (Bio-Rad, Hercules, CA) [21].

Additionally, a sample of each crude CFE (15 ml) was sequentially size fractionated with Microsep^™^ Advance Centrifugal Devices (Pall Corporation, Port Washington, NY) with the molecular weight cut-offs (MWCO) of 100K, 30K, 10K, and 3K. This separated the CFE into five fractions containing proteins with approximate molecular weight of >300 kDa, 90–180 kDa, 30–90 kDa, 10–20 kDa, and <10 kDa, respectively. The fractionated solutions were lyophilized and dissolved in MQ water to achieve the final protein concentration of approximately 20 mg/ml to match that of the crude extract. All samples were stored at -80°C until the antibacterial assays.

### Protein denaturation

To denature proteins, 5 ml of the lyophilized crude extract of each treatment was heated to 100°C for five minutes as previously described [12]. Trypsin digestion was performed as follows. To 100 μl of lyophilized crude extract for each treatment, 5 μl of 200 mM dithiothreitol (DTT) in 100 mM NH_4_HCO_3_ was added and incubated for 30 min at room temperature. Then, 4 μl of the 1M iodoacetamide alkylating reagent was added to the sample, mixed, and incubated for 45 min at room temperature. Finally, 50 μl of trypsin (0.2 μg/μl in 100 mM NH_4_HCO_3_) was added to the sample and incubated overnight at 37°C.

### Termite hemolymph collection and hindgut extraction

Hemolymph was immediately drawn (~0.05–0.1 μl/individual) from surface sterilized termites by inserting a sterile insect needle into the dorsal intersegmental membrane of cold-immobilized insects. Any sample contaminated with the gut or fat was discarded. The extracted hemolymph (~200–400 μl) was transferred to a microcentrifuge tube containing 1 ml of 20 mM Tris-HCl, 20 mM NaCl (pH = 7.5) buffer and kept on ice. Hindguts for the same individuals were separated and rinsed in 5 ml of 20 mM Tris-HCl, 20 mM NaCl (pH = 7.5) buffer before being homogenized in 1ml of buffer on ice. The hemolymph and gut extracts were centrifuged at 12,000 rpm at 4°C for 5 min to acquire the cell-free samples. The protein concentration of each sample was measured and was adjusted to the final concentration of 25 mg/ml by either dilution or concentration following lyophilization.

### Antibacterial assay

Antibacterial activity of each extract against a panel of selected bacteria (Table 1) was determined using a modified inhibition zone assay as previously described [12]. For every assay, each bacterium was freshly grown from a frozen stock. Briefly, a colony from a freshly streaked plate was inoculated into LB medium, grown overnight at 37°C with shaking at ~220 rpm, subcultured the next day into fresh LB medium and grown at 37°C with shaking at ~220 rpm to early log-phase of growth (OD_600_ = 0.3 ± 0.05), and diluted to ~2.5 × 10^7^ CFU/ml. The antibacterial activities of crude extracts were examined by placing eight sterilized filter paper disks (5 × 5 mm) uniformly on the bacterial lawn in each plate. The paper disks were treated with one of the following eight samples, respectively: 20 μl of three crude extracts (naïve, *P*. *aeruginosa*-challenged, and MRSA-challenged) at a concentration of approximately 20 mg/ml (= 400 μg/disk), 20 μl of three heat-treated crude extracts (naïve, *P*. *aeruginosa*-challenged, and MRSA-challenged), 1 μl of ampicillin (= 25 μg/disk) as the positive control, and 20 μl of 80 mM Tris-HCl, 80 mM NaCl buffer as the negative control. The antibacterial activities of size-fractionated extracts, hemolymph, and gut extract were determined using the same assay. All plates were incubated at 37°C for 24 h to allow bacterial growth and the zones of clearing were measured. Every assay was repeated three times, each with 3 replicates, to acquire an N of 9 for each treatment.

**Table 1:** List of the tested bacteria.

Bacterium	Gram Stain	Multi-Drug Resistant	Source or Reference
*Staphylococcus aureus*	**+**	No	ATCC 12600 via Robert Miller
Methicillin-resistant *Staphylococcus aureus* (MRSA)	**+**	Yes	James Barbaree
*Streptococcus pyogenes*	**+**	No	ATCC 19615 via Robert Miller
*Pseudomonas aeruginosa* (PAO1)	**-**	Yes	[22]
*Escherichia coli* O157:H7 (CDC B1409-C1)	**-**	No	ATCC 43889
*E*. *coli* K-12 (MG 1655)	**-**	No	ATCC 700926
*Salmonella enterica* serovar Typhimurium (LT2)	**-**	No	[23] via Jorge Escalante-Semerena
*Acinetobacter baumannii* (AYE)	**-**	Yes	ATCC BAA-1710 [24]

### Statistical analysis

The diameters (D; mm) of inhibition zones were compared using the ANOVA and Tukey’s method (PROC GLM; α = 0.05; SAS 9.2) to determine all possible pairwise differences among treatments. The ANOVA (PROC GLM; α = 0.05; SAS 9.2) was used to determine the difference in activities of the same treatment on each tested bacterium.

### Gel electrophoretic analysis of proteins

Protein profiles of the MWCO of 30K and 100K fractionated samples of termite CFE were analyzed by polyacrylamide gel electrophoreses (PAGE) including both non-denaturing Native PAGE and denaturing SDS-PAGE. Approximately 20 μg of protein samples were loaded per lane. The protein ladders for Native PAGE (14–500 kDa) and SDS-PAGE (10–250 kDa) were purchased from Sigma-Aldrich (St Louis, MI) and Life Technologies (Green Island, NY), respectively. In addition, non-size fractionated hemolymph samples were analyzed on SDS-PAGE. The proteins on PAGE were stained with Coomassie brilliant blue R-250 (Bio-Rad, Hercules, CA) for visualization.

Two-dimensional gel electrophoretic analysis of the proteins in hemolymph was performed by the Kendrick Labs, Inc. (Madison, WI). Approximately 200 μg of total protein per sample was used to separate the proteins between pI of 3 to 10 for the first dimension and molecular weight of 14–220 kDa for the second dimension. Duplicate gels were run for each sample, stained with Sypro^®^Ruby (Bio-Rad), and scanned on a Typhoon FLA 9000 scanner (GE Healthcare, Piscataway, NJ). A total of 493 spots were analyzed using Progenesis SameSpots software (version 4.5, 2011, TotalLab, UK) and Progenesis PG240 software (version 2006, TotalLab, UK). The quantity of each spot was calculated as spot percentages (individual spot density divided by total density of all measured spots). The quantity differences between MRSA-challenged versus naïve and *P*. *aeruginosa*-challenged versus naïve were analyzed using two-sample t-test.

## Results

### Broad-spectrum constitutive antibacterial activity of *R*. *flavipes*

In order to understand the biological range of antibacterial activity of *R*. *flavipes* CFE, we tested a panel of non-MDR and MDR human bacterial pathogens for their susceptibility. Table 1 shows the panel of bacteria used in this study which includes both gram-positive and gram-negative bacteria. The CFE prepared from the whole body of naïve termite displayed significant inhibitory activity against the five non-MDR bacteria (*Staphylococcus aureus*, *Streptococcus pyogenes*, *Escherichia coli* O157:H7, *Salmonella enterica* serovar Typhimurium, and *E*. *coli* K-12) but it was inactive against the three MDR pathogens (methicillin resistant *S*. *aureus* or MRSA, *Pseudomonas aeruginosa*, and *Acinetobacter baumannii*) (Table 2). Of the five susceptible bacteria, the strongest inhibitory effect was against *S*. *aureus*, followed by *E*. *coli* O157:H7 and *S*. Typhimurium. The weakest activity was against *S*. *pyogenes*. As expected, ampicillin inhibited the growth of all but the three MDR pathogens. We observed no obvious correlation between Gram-staining and the effectiveness of the termite CFE on growth inhibition. The antibacterial activity of termite CFE disappeared completely when it was heat-denatured (Table 2) or treated with trypsin (data not shown). These data suggest that the antibacterial activity of *R*. *flavipes* is likely to be proteinaceous in nature.

**Table 2:** Antibacterial activity of CFE of naïve termites.

Bacteria			Termite crude extract (400 μg)	Ampicillin (25 μg)	Heat-treated crude extract	Control*
**Non-infectious**		*E*. *coli*	9.41 ± 0.37^d^	8.49 ± 0.33^e^	0	0
**Infectious**	**Non-MDR**	*S*. *aureus*	16.16 ± 0.73^a^	27.99 ± 0.4^a^	0	0
		*S*. *pyogenes*	9.14 ± 0.30^d^	16.41 ± 0.36^b^	0	0
		*E*. *coli* O157:H7	14.51 ± 0.57^b^	11.28 ± 0.24^d^	0	0
		*S*. Typhimurium	10.42 ± 0.50^c^	12.39 ± 0.28^c^	0	0
	**MDR**	MRSA	0	0	0	0
		*P*. *aeruginosa*	0	0	0	0
		*A*. *baumannii*	0	0	0	0

Different letters within a column indicate significant difference in inhibition zone diameters at significance level of α = 0.05 (ANOVA, *F*_4, 40(CrudeExtract)_ = 1854.68, *P* < 0.0001; *F*_6, 56(Ampicillin)_ = 2960.78, *P* < 0.0001). The experiment was performed three independent times with triplicate samples for a total N of 9 for each treatment. The diameter of zone of inhibition was measured following 24 h incubation at 37°C.

*Control: 80 mM Tris-HCl, 80 mM NaCl buffer.

### MDR-induced alteration in antibacterial activities of *R*. *flavipes*

**Fig 1:**
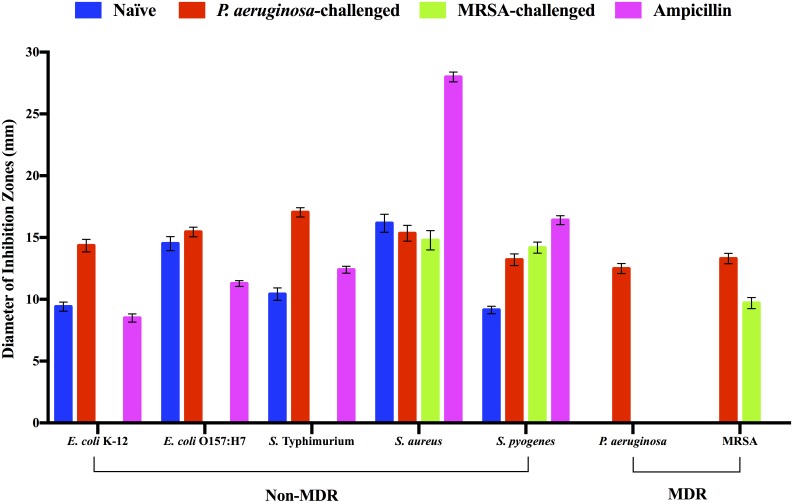
Antibacterial activities of cell free extracts of *R*. *flavipes*. Approximately 400 μg of CFE of the naïve, *P*. *aeruginosa-*challenged, and MRSA-challenged *R*. *flavipes* was applied respectively to each filter disk on a bacterial lawn. The zone of inhibition of growth was measured following incubation for 24 hours at 37°C. Ampicillin was used as the positive control at 25 μg per filter disk and 20 μl of buffer was used as the negative control. The data shown are a compilation of three independent experiments done in triplicate for a total N of 9 per sample (ANOVA, *F*_27, 224 = 2635.47_, *P* < 0.0001). Data for *A*. *baumannii* are not shown because the termite extracts were ineffective against the bacterium.

In addition to the constitutive antibacterial activity, we determined that *R*. *flavipes* possesses inducible antibacterial activities. Feeding termites with heat-killed *P*. *aeruginosa* or MRSA altered their antibacterial activities and stimulated specific anti-MDR activity as illustrated in Fig 1. In both cases, the termites produced new antibacterial activity that was effective against the inducer bacterium. Specifically, termite challenged by heat-killed *P*. *aeruginosa* produced activity against both *P*. *aeruginosa* and MRSA while maintaining or slightly increasing antibacterial activity against bacteria listed in Table 1. Termites challenged by heat-killed MRSA produced anti-MRSA activity while maintaining activity against two non-MDR Gram-positive pathogens. Interestingly, antibacterial activity against Gram-negative bacteria listed in Table 1 was completely abolished in MRSA-challenged termites (Fig 1). Neither *P*. *aeruginosa* nor MRSA induced antibacterial activity against *A*. *baumannii* in *R*. *flavipes* (data not shown).

### Size fractionation of antibacterial activities

**Fig 2:**
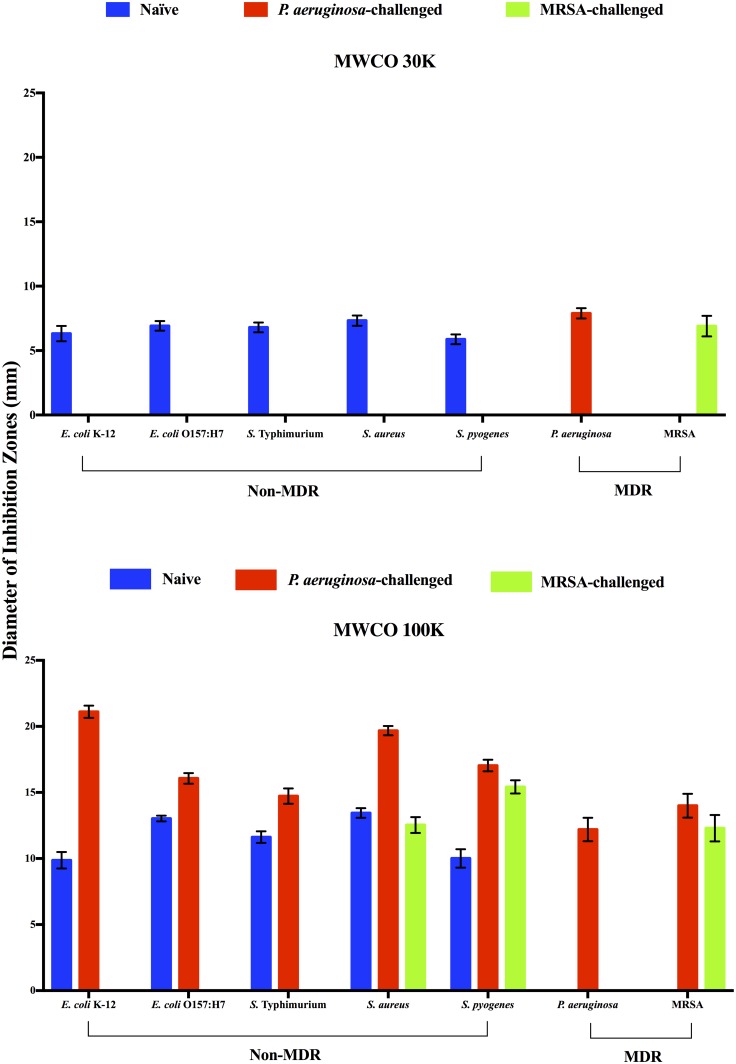
Antibacterial activity of size-fractionated cell free extracts of *R*. *flavipes*. Antibiotic activity was measured as diameter of inhibition zones caused by respective application of approximately 400 μg of size-fractionated CFE from the naïve, *P*. *aeruginosa-*challenged, and MRSA-challenged *R*. *flavipes* on a bacterial lawn. The data shown are a compilation of three independent experiments done in triplicate for a total N of 9 per sample (ANOVA, *F*_27, 224 = 2635.47_, *P* < 0.0001). (A) MWCO 30K (90–180 kDa) fraction. (B) MWCO 100K (>300 kDa) fraction. Data for fractions of <10 kDa, 10–20 kDa, 30–90 kDa are not shown because they did not demonstrate antibacterial activities.

We previously demonstrated that multiple fractions of size-fractionated *R*. *flavipes* CFE had antibacterial activity against the soil-borne entomopathogenic *B*. *subtilis* [12]. This indicated that *R*. *flavipes* produced multiple proteins with anti-*B*. *subtilis* activity. In continuing our characterization, we tested size-fractionated CFE from the naïve, *P*. *aeruginosa* challenged, and MRSA challenged *R*. *flavipes* against the panel of bacteria listed in Table 1. We determined that all antibacterial activities of three groups of termites against this panel of bacteria were contained in fractions with proteins larger than 90 kDa in molecular weight (MWCO filters of 30K and 100K; S1 Fig). However, many of these large proteins appeared to be composed of smaller subunits because they migrated as proteins of 25–90 kDa on denaturing SDS-PAGE (S2 Fig). In all three groups of termites, the MWCO 100K fractions containing proteins of >300 kDa demonstrated greater activity against the susceptible bacteria than the MWCO of 30K fractions with 90–180 kDa proteins (Fig 2). These data support our previous finding that *R*. *flavipes* possesses multiple proteins with antibacterial activity.

The *P*. *aeruginosa*-induced antibacterial activities against the seven susceptible bacteria were all due to the protein fraction from the MWCO 100K filter except against the inducer bacterium. The anti-*P*. *aeruginosa* activity was present in both the MWCO 100K and 30K fractions, indicating the possibility of induction of multiple anti-*P*. *aeruginosa* proteins. In addition, the *P*. *aeruginosa-*challenged termites exhibited more effective antibacterial activity in the MWCO 100K protein fraction against those non-MDR bacteria than did the naïve termites. The increased activity in the MWCO 100K fraction was especially evident against *E*. *coli* K-12 and *S*. *aureus*, indicating that the induction of antibacterial activity by *P*. *aeruginosa* was independent of the Gram staining-based classification of bacteria. Interestingly, *P*. *aeruginosa* induced greater anti-MRSA activity in the MWCO 100K fraction than did MRSA.

Similar to *P*. *aeruginosa* induction, the MRSA-induced antibacterial activity was contained in the MWCO 100K fraction with the exception of anti-MRSA activity which was present in both the MWCO 30K and 100K fractions.

### Origin of *R*. *flavipes* antibacterial activity

**Fig 3:**
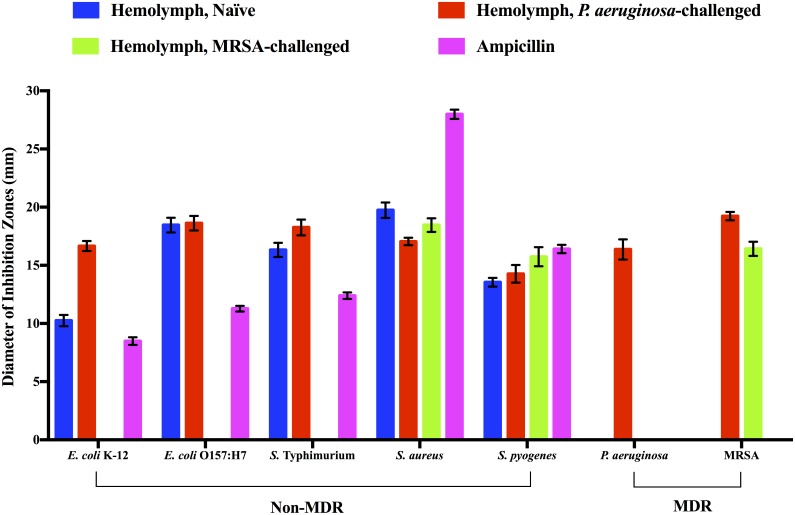
Antibacterial activity of *R*. *flavipes* hemolymph. Approximately 400 μg of hemolymph extract from the naïve, *P*. *aeruginosa-*challenged, and MRSA-challenged *R*. *flavipes* was applied respectively on a bacterial lawn. The zone of inhibition was measured. Ampicillin was used as the positive control at 25 μg per filter disk. The data shown are a compilation of three independent experiments done in triplicate for a total N of 9 per sample (ANOVA, *F*_41, 336_ = 9762.44, *P*<0.0001).

In order to determine whether the antibacterial activities were of termite origin or of the gut microbiota, we prepared extracts from the hindgut and compared the activity to the hemolymph. As illustrated in Fig 3, the antibacterial activity against our panel of bacteria were only observed in the hemolymph of the naïve, *P*. *aeruginosa-*challenged, or MRSA-challenged. Hindgut extracts from the same termites showed no perceived antibacterial activities (data not shown). The antibacterial activity profile of the hemolymph resembled that of the whole-body extract shown in Fig 1. Our data suggest that both constitutive and inducible antibacterial activities of *R*. *flavipes* are likely of termite origin and reside in hemolymph.

### Protein profiles of termite hemolymph

We analyzed the hemolymph protein profiles of naïve, *P*. *aeruginosa-*challenged, and MRSA-challenged termites via both one-dimensional and two-dimensional gel electrophoreses to identify differentially expressed proteins. On one-dimensional 8% Native PAGE, we observed very little difference between the naïve, MRSA-challenged, and *P*. *aeruginosa-*challenged termites for both 100K and 30K MWCO fractionated samples (S1 Fig). On denaturing 8% SDS-PAGE, we observed subtle differences between the three samples and between the 100K and 30K MWCO fractionated samples (S2 Fig). Specifically, there was a slight upregulation of ~50 kDa, ~110 kDa and ~150–200 kDa proteins, and a slight downregulation of ~35 kDa, ~55 kDa proteins in MRSA-challenged termites. For *P*. *aeruginosa-*challenged termites, we observed a slight upregulation of ~80 kDa, ~50 kDa, and ~35 kDa proteins and a slight downregulation of >250 kDa and ~60 kDa proteins. When the hemolymph samples were analyzed on 8% SDS-PAGE, we observed two abundant proteins between ~60–85 kDa that appeared to be conserved among the naïve, *P*. *aeruginosa-*challenged, and MRSA-challenged termites. We also observed some differences between three samples but especially for *P*. *aeruginosa*-challenged termites in which we saw disappearance of a prominent band of ~250 kDa and appearance of ~35 kDa, ~50 kDa, and ~65 kDa (S3 Fig).

**Fig 4:**
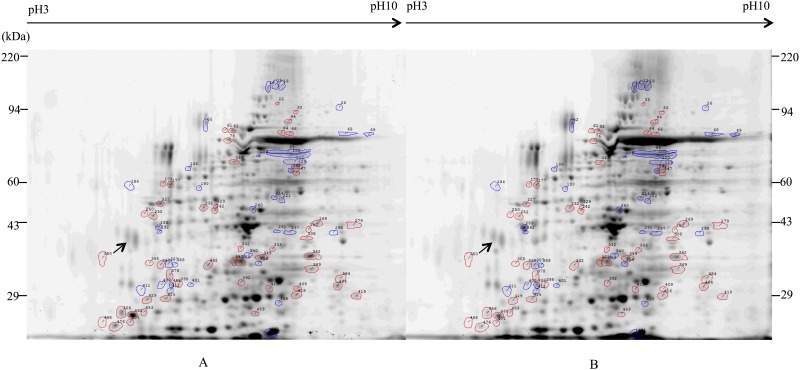
Two-dimensional electrophoretic analysis of hemolymph proteins from naïve and *P*. *aeruginosa*-challenged *R*. *flavipes*. Approximately 200 μg of hemolymph proteins were separated by two-dimensional gel electrophoresis and visualized with Sypro^®^Ruby. Blue circles indicate protein spots that are upregulated in *P*. *aeruginosa* challenged termite while red circles indicate protein spots that are downregulated. (A) Naïve termites. (B) *P*. *aeruginosa-*challenged termites.

**Fig 5:**
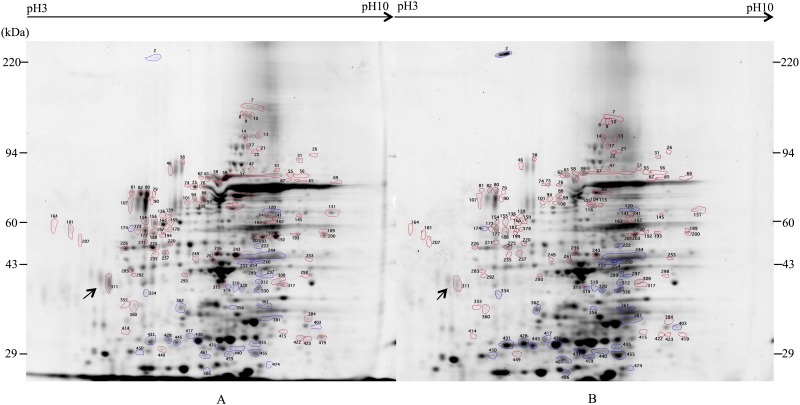
Two-dimensional electrophoretic analysis of hemolymph proteins from naïve and MRSA-challenged *R*. *flavipes*. Approximately 200 μg of hemolymph proteins were separated by two-dimensional gel electrophoresis and visualized with Sypro^®^Ruby. Blue circles indicate protein spots that are upregulated in MRSA challenged termite and red circles indicate protein spots that are downregulated. (A) Naïve termites. (B) MRSA*-*challenged termites.

It was clear from our analyses that termite hemolymph was too complex to be accurately analyzed via one-dimensional PAGE. Thus, we performed two-dimensional electrophoretic analyses of the naïve, *P*. *aeruginosa-*challenged, and MRSA-challenged termite hemolymphs. Based on our data, the termite hemolymph contains approximately 493 proteins that are visible on two-dimensional gel stained with Sypro^®^Ruby. A comparison of the hemolymph proteins between the naïve (Fig 4A) and the *P*. *aeruginosa-*challenged termites (Fig 4B) indicated that 38 proteins were differentially expressed at least 2.5-fold (P<0.05). Of these, 18 proteins were upregulated and 20 proteins were downregulated (S1 Table). A comparison of the naïve termites (Fig 5A) and the MRSA-challenged termites (Fig 5B) showed that 65 proteins were differentially expressed at least 2.5-fold (P<0.05). Of these, 11 proteins were upregulated and 54 proteins were downregulated (S2 Table).

In *P*. *aeruginosa*-challenged termites, the highest upregulated protein (approximately 11-fold increase) had MW of approximately 37 kDa. The 20 downregulated proteins displayed no discernable pattern in size. In MRSA-challenged termites, the majority of upregulated proteins (11 spots) had MW of approximately 18 to 58 kDa while downregulated proteins (53 spots) were all larger than 28 kDa. The alteration of hemolymph protein profile in response to bacterial challenge support our assertion that *R*. *flavipes* contains both constitutive and inducible antibacterial proteins.

We compared some of the differentially expressed hemolymph proteins to previously identified insect immune proteins based on their relative pI and MW. The results are shown in Tables 3 and 4 for *P*. *aeruginosa*-induced and MRSA-induced proteins, respectively. Among the *P*. *aeruginosa*-induced proteins, six proteins of approximately 30–55 kDa proteins had similar pI and MW to previously identified insect immune proteins. We did not find any insect immune proteins of ≥90 kDa in the literature that had similar pI and MW as the spots we identified (S1 Table). Among the MRSA-induced proteins, we found three proteins of approximately 29–60 kDa in MW that had similar pI and MW to previously identified insect immune proteins. Similar to *P*. *aeruginosa-*induced proteins, we did not find any insect immune proteins of ≥90 kDa in the literature that had similar pI and MW as the spots we identified in our MRSA-challenged hemolymph samples (S2 Table).

**Table 3:** Comparison of *P*. *aeruginosa*-induced termite hemolymph proteins to insect immune proteins.

Spot	Fold change	pI	MW (Da)	Insect immune proteins	pI	MW (Da)	Reference
491	3.7	7.1	17,661	Antibacterial peptide (*Bombyx mori*)	6.8	18,777	[25]
491	3.7	7.1	17,661	Attacin-like immune protein	7.0	17,588	http://www.ncbi.nlm.nih.gov/protein/AHB11276.1
491	3.7	7.1	17,661	Gloverin 4 (*Bombyx mori*)	6.8	18,777	[25]
358	7.2	5.7	36,011	Toll (*Sitophilus oryzae*)	5.2	37,609	[26]
400	2.9	5.6	32,736	Immune-related Hdd13 (*Hyphantria cunea*)	5.7	29,691	[27]
402	3.1	5.7	32,692	Antimicrobial protein 6 Tox precursor (*Galleria mellonella*)	5.7	32,542	[28]
358	7.2	5.7	36,011	Immune-related Hdd1 (*Hyphantria cunea*)	5.2	35,611	[27]
283	3.2	5.6	42,081	Termite GNBPs (*Nasutitermes coniger*)	5.6	42,323	[13]
214	3.8	7.2	52,154	β-1,3-glucan-binding protein/Gram negative bacteria-binding protein precursor (*Hyphantria cunea*)	7.1	53,014	[29]

The pI and the MW of termite hemolymph proteins are based on the Kendrick Labs’ analysis of the two-dimensional gels. We used arbitrary cutoff values of ≤0.5 in pI and ≤5 kDa between our hemolymph proteins and previously reported insect proteins to determine potential relationship.

**Table 4:** Comparison of MRSA-induced termite hemolymph proteins to insect immune proteins.

Spot	Fold change	pI	MW (Da)	Insect immune proteins	pI	MW (Da)	Reference
486	7.7	6.6	18,674	Antibacterial peptide (*Bombyx mori*)	6.4	18,821	[25]
486	7.7	6.6	18,674	Gloverin 2 (*Bombyx mori*)	6.4	18,821	[25]
486	7.7	6.6	18,674	Gloverin 4 (*Bombyx mori*)	6.8	18,777	[25]
428	3	6.0	26,359	Attacin-E (*Hyalophora cecropia*)	6.1	25,438	[30]
450	2.5	5.7	27,591	Possible antimicrobial peptide (*Bombyx mori*)	6.3	27,469	[31]
440	3	7.1	29,320	Phospholipase A2B precursor (*Tribolium castaneum*)	7.6	29,425	[32]
440	3	7.1	29,320	Spz1A, partial (*Manduca sexta*)	7.6	29,195	[33]
440	3	7.1	29,320	Scolexin A (*Manduca sexta*)	7.0	30,373	[34]
254	6.3	7.2	44,637	Putative hemolin (*Hyphantria cunea*)	6.7	46,119	[27]
174	2.7	5.5	58,639	Gram-negative bacteria binding protein 3 (*Drosophila melanogaster*)	6.0	55,322	[35]

The pI and the MW of termite hemolymph proteins are based on the Kendrick Labs’ analysis of the two-dimensional gels. We used arbitrary cutoff values of ≤0.5 in pI and ≤5 kDa between our hemolymph proteins and previously reported insect proteins to determine potential relationship.

## Discussion

The current study demonstrates that the eastern subterranean termite, *R*. *flavipes*, produces innate and inducible antibacterial activities that are effective against several human pathogens including two MDRs. The list of pathogens found to be susceptible to naïve *R*. *flavipes’* extract includes bacteria that cause gastroenteritis (*E*. *coli* O157:H7 and *S*. Typhimurium), and common opportunistic and nosocomial pathogens (*S*. *aureus* and *S*. *pyogenes*). The presence of innate antibacterial proteins in *R*. *flavipes* parallels the results found in a fungus-growing termite *Pseudacanthotermes spiniger* and a pacific dampwood termite *Zootermopsis angusticollis* [8, 16]. Recent analysis of *Z*. *nevadensis* genome revealed that multiple effector immune response genes, including GNBPs, attacin, diptericin and termicin, are encoded in this termite [36]. It is likely that similar products may be found in the hemolymph of *R*. *flavipes* since there is a high degree of genetic conservation among termites. Interestingly, in contrast to other studies, including our own study demonstrating fractions of MWCO of 3K and 10K of naïve *R*. *flavipes* CFE inhibiting growth of the entomopathogenic *B*. *subtilis* [12], all of the activities against the infectious human pathogens we identified in this study were larger than ≥90 kDa contained within the MWCO of 30K and 100K fractions. However, based on our SDS-PAGE analyses, some of the larger proteins appear to be multisubunit complexes as they denatured into smaller proteins.

Constitutive defense mechanisms of insects usually rely on the response of hemocytes and several enzyme cascades such as phenoloxidase to defend against potential pathogens [1]. Although the exact identity or the molecular mechanisms of innate antibacterial activities of *R*. *flavipes* have yet to be characterized, we identified the hemolymph as the source of these activities. This suggests that the antibacterial activities seen in naïve termites are a part of *R*. *flavipes’* constitutive immune system.

In addition to the constitutive antibacterial activities against several non-MDR human pathogens, we successfully demonstrated induction of specific activities using two MDR human pathogens as antagonists. Induction of antimicrobial activity in insects is not new. In 2012, Hussain et al. [17] described a low level induction of antibacterial activity in the Formosan subterranean termite, *C*. *formosanus*, when the termite was immersed in suspensions of an entomopathogenic fungus (*M*. *anisopliae*) or several bacteria. However, of the bacteria used in the study (*S*. *aureus*, *B*. *thuringiensis*, *E*. *coli*, and *Ralstonia solanacearum*), only *B*. *thuringiensis*, which produces anti-insect toxins, induced antimicrobial activity. Interestingly, our results suggest that both Gram-positive and Gram-negative bacteria can be strong inducers, and antibacterial responses can be observed 24 h after heat-killed bacteria challenge. This is supported by a recent study showing that specific combinations of immune genes in *R*. *flavipes* were expressed in responding to the exposure of various infective fungal spores [37]. Other studies have demonstrated specific response of the American cockroach, *Periplaneta Americana* [38], and the bumblebee, *Bombus terrestris* [39], to Gram-negative and Gram-positive bacteria. However, our study is the first one to demonstrate induction of specific activities in termite against MDR human pathogens.

The pattern of induced antibacterial activity based on the bacterium used as the antagonist suggests a complex phenomenon. *P*. *aeruginosa-*challenge induced anti-*P*. *aeruginosa* activity while maintaining or slightly increasing antibacterial activities across a broad spectrum of bacteria tested. In contrast, MRSA-challenge induced the activity against the antagonist while maintaining or slightly increasing activity only against two Gram-positive bacteria, *S*. *aureus* and *S*. *pyogenes*. Thus, these induction patterns appear to reduce the possibility of peptidoglycan or lipopolysaccharide serving as the major antagonist for *R*. *flavipes* against these bacteria. Both *P*. *aeruginosa* and MRSA possess peptidoglycan while only *P*. *aeruginosa* possesses lipopolysaccharide. Interestingly, heat-killed *S*. *aureus* failed to induce anti-MRSA activity (data not shown), thereby lending support that peptidoglycan is unlikely to be the inducer. Thus, MRSA likely invoked some immune response in *R*. *flavipes* that is specific to MRSA that is missing in *S*. *aureus* (*i*.*e*. staphyloccal cassette chromosome *mec* or SCC*mec*) [40, 41], while *P*. *aeruginosa* invoked a response that is effective against a multitude of bacteria.

Termites contain a complex microbiota in their alimentary tract that could have contributed to the observed inducible antibacterial activity [1, 42]. We suspected that the microbial symbionts from the hindgut, as well as termite immune proteins, could be the source of the antibacterial activity [43, 44] in our assays. However, our comparative analysis of the hemolymph versus the hindgut localized all of the observable antibacterial activity to the hemolymph, suggesting termite cells as the origin of antibacterial activity. Our finding agrees with a previous study that reported the inability of oral ingestion of fungal spores and bacteria to induce innate gut defenses in *R*. *flavipes* [45]. The authors of that study speculated a lack of inducible genes being present on the microarray or weak innate defense in this termite.

Several antimicrobials have been identified from termites including termicin, spinigerin, lysozymes, tGNBPs, and transferrin [8, 11, 14–16, 46]. However, given the small molecular weight of most these previously identified peptides/proteins ≤15 kDa), it is likely that we have discovered novel proteins or protein complexes, with antibacterial activity since most of our activity is limited to proteins of ≥90 kDa. This finding agrees with previous studies demonstrating various protein complexes functioning as antimicrobial effectors with large molecular weight (>150 kDa) in insects, plants, and microorganisms [47–51]. Because our hemolymph samples were CFE and the induced antibacterial proteins appeared to be involved in humoral reaction, it is possible that these proteins were induced by Relish [15]. Based on recent identification of differentially expressed proteins involved in stress response, immune signaling, biosynthesis and other functions in *R*. *chinensis* following an entomopathogenic fungal infection [52], mechanisms of insect immunity regulation appear to be complex.

Our two-dimensional gel analyses indicate that some of the upregulated proteins found in the induced termite hemolymph may be similar to previously identified insect immune proteins as listed in Tables 3 and 4. To compare our proteins to known insect immune proteins, we arbitrary designated a cutoff point of ≤0.5 in pI and ≤5000 Da in MW. However, given that mobility of a protein on IEF and SDS-PAGE can be affected by various factors including protein processing and modification, it is possible that our comparison is not comprehensive. We are currently in the process of identifying the differentially expressed proteins in our hemolymph samples to better understand the induction of the immune response in *R*. *flavipes* to bacterial challenge.

## Supporting Information

S1 Fig 8% Non-denaturing PAGE analysis of MWCO of 30K and 100K size-fractionated samples of termite CFE. Lanes 1–4: Protein ladders of α-lactalbumin from bovine milk, albumin from bovine serum, albumin from chicken egg white, and urease from jack bean, respectively. Lanes 5–7: MWCO of 100K fractions from *P*. *aeruginosa-*challenged, MRSA-challenged, and naïve termites, respectively. Lanes 8–10: MWCO of 30K fractions from *P*. *aeruginosa-*challenged, MRSA-challenged, and naïve termites, respectively.
(TIF)

S2 Fig 8% SDS-PAGE analysis of MWCO of 30K and 100K size-fractionated samples of termite CFE. Lane 1: Protein ladder (10–250 kDa); Lanes 2–4: MWCO of 100K fractions from *P*. *aeruginosa-*challenged, MRSA-challenged, and naïve termites, respectively. Lanes 5–7: MWCO of 30K fractions from *P*. *aeruginosa-*challenged, MRSA-challenged, and naïve termites, respectively.
(TIF)

S3 Fig 8% SDS-PAGE analysis of hemolymph proteins. Lane 1: Protein Ladder (10–250 kDa); Lanes 2–4: Hemolymph proteins from naïve, MRSA-challenged, and *P*. *aeruginosa-*challenged termites, respectively.
(TIF)

S1 Table Differentially expressed hemolymph proteins in *P*. *aeruginosa*-challenged termites with at least 2.5-fold change. The pI and the MW of termite hemolymph proteins are based on the Kendrick Labs’ analysis of the two-dimensional gels.
(DOCX)

S2 Table Differentially expressed hemolymph proteins in MRSA-challenged termites with at least 2.5-fold change. The pI and the MW of termite hemolymph proteins are based on the Kendrick Labs’ analysis of the two-dimensional gels.
(DOCX)
