# Supplementary figures and images for: Characterization of Antibacterial Activities of Eastern Subterranean Termite, Reticulitermes flavipes, against Human Pathogens
Source: PLoS One. 2016 Sep 9;11(9):e0162249. doi: 10.1371/journal.pone.0162249 (PMC5017719; doi:10.1371/journal.pone.0162249)

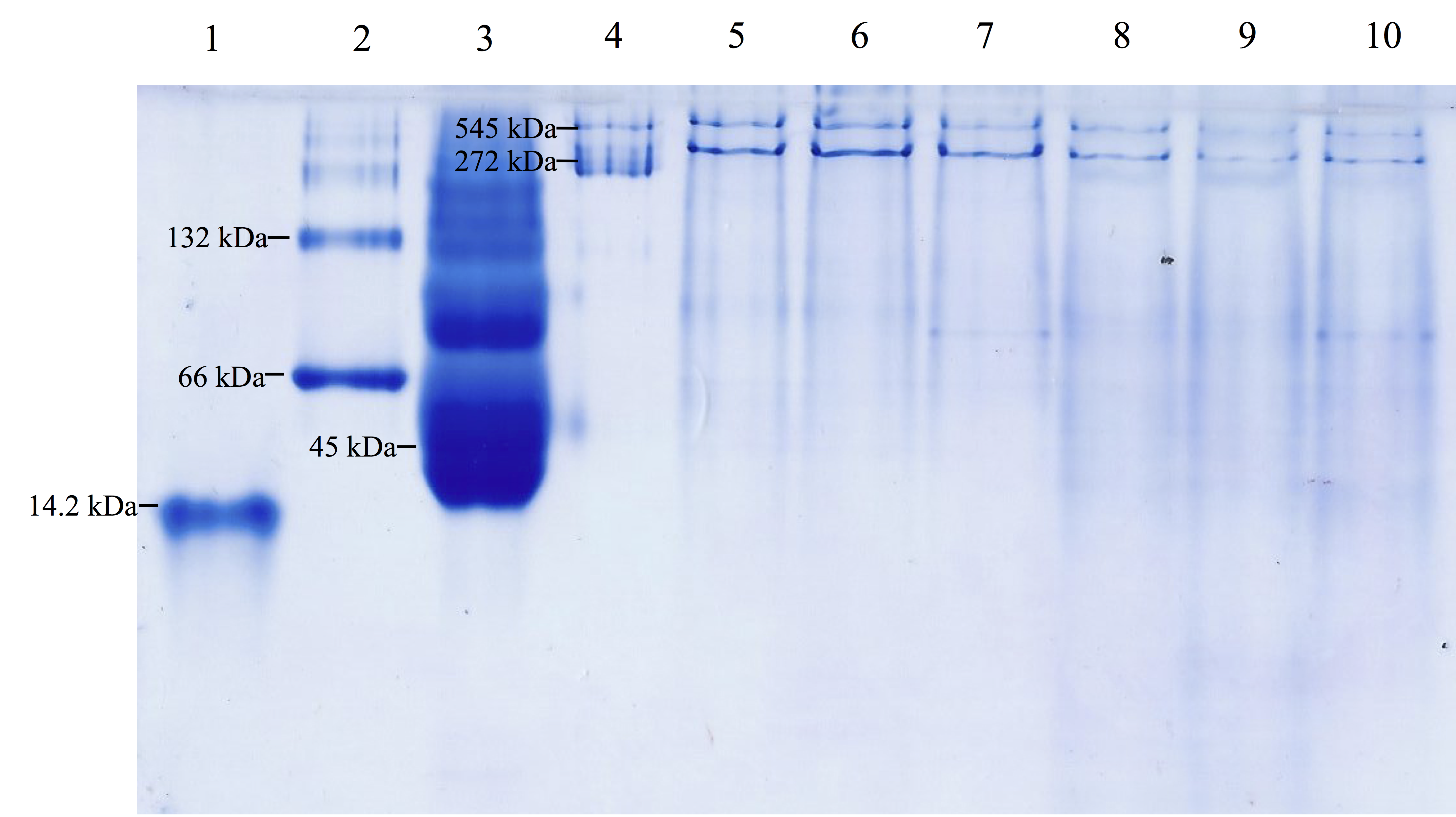

Supplement: S1 Fig — Lanes 1–4: Protein ladders of α-lactalbumin from bovine milk, albumin from bovine serum, albumin from chicken egg white, and urease from jack bean, respectively. Lanes 5–7: MWCO of 100K fractions from P. aeruginosa-challenged, MRSA-challenged, and naïve termites, respectively. Lanes 8–10: MWCO of 30K fractions from P. aeruginosa-challenged, MRSA-challenged, and naïve termites, respectively. (TIF) [file pone.0162249.s001.tif]

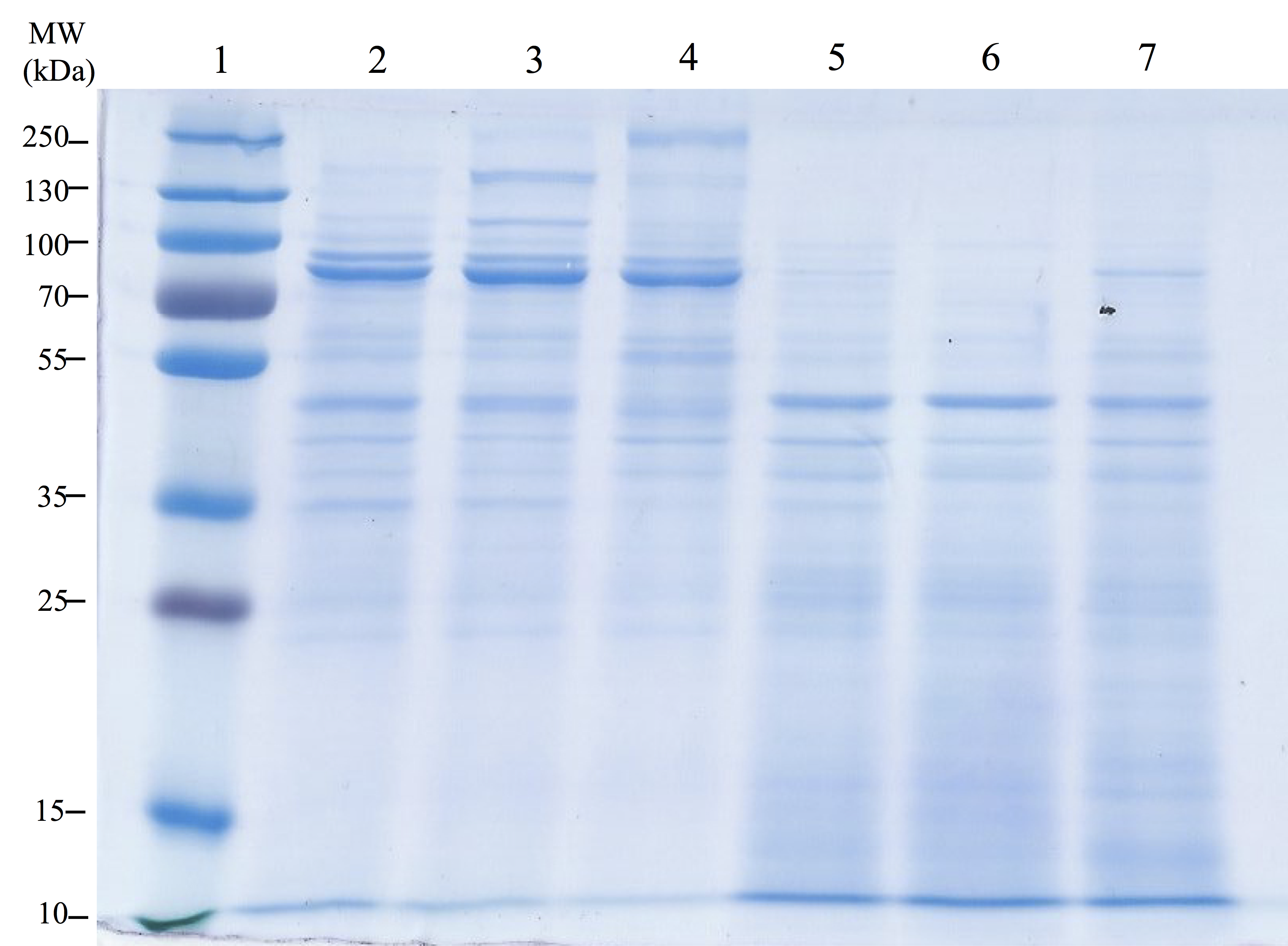

Supplement: S2 Fig — Lane 1: Protein ladder (10–250 kDa); Lanes 2–4: MWCO of 100K fractions from P. aeruginosa-challenged, MRSA-challenged, and naïve termites, respectively. Lanes 5–7: MWCO of 30K fractions from P. aeruginosa-challenged, MRSA-challenged, and naïve termites, respectively. (TIF) [file pone.0162249.s002.tif]

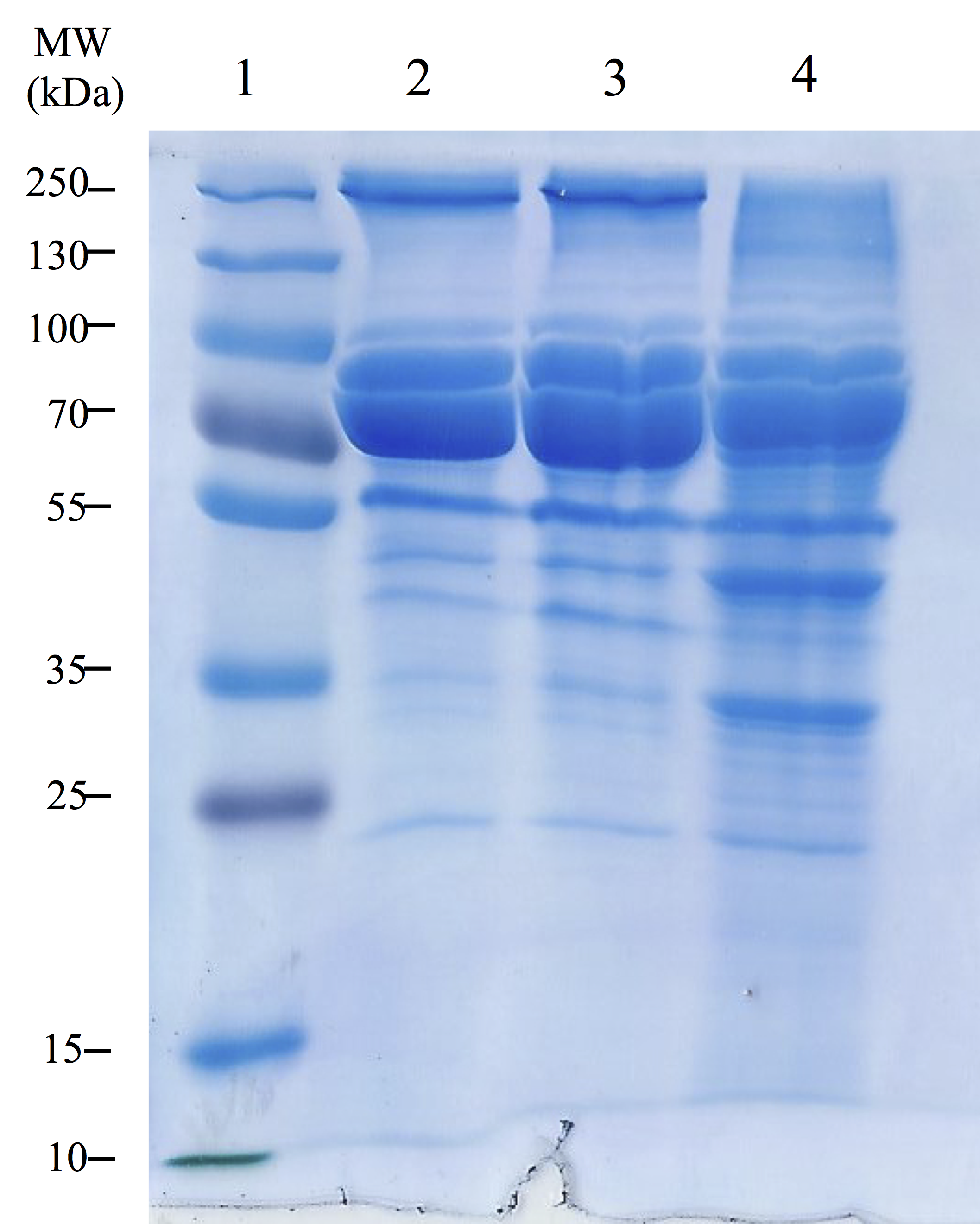

Supplement: S3 Fig — Lane 1: Protein Ladder (10–250 kDa); Lanes 2–4: Hemolymph proteins from naïve, MRSA-challenged, and P. aeruginosa-challenged termites, respectively. (TIF) [file pone.0162249.s003.tif]
