# Supplementary material for: Characterization of Antibacterial Activities of Eastern Subterranean Termite, Reticulitermes flavipes, against Human Pathogens
Source: PLoS One. 2016 Sep 9;11(9):e0162249. doi: 10.1371/journal.pone.0162249 (PMC5017719; doi:10.1371/journal.pone.0162249)
Supplement: S1 Table — The pI and the MW of termite hemolymph proteins are based on the Kendrick Labs’ analysis of the two-dimensional gels. (DOCX) [file pone.0162249.s004.docx]

**S1 Table.**

| **Spot #** | **pI** | **MW** | ***P. aeruginosa* vs Naïve Difference** | **T-test of *P. aeruginosa* vs Naïve** |
| --- | --- | --- | --- | --- |
|  |  | **(Da)** |  |  |
| **491** | 7.1 | 17,661 | 3.7 | 0.017 |
| **471** | 5.2 | 22,326 | -2.8 | 0.009 |
| **468** | 4.6 | 22,510 | -6 | 0.013 |
| **460** | 5.2 | 25,510 | -2.6 | 0.012 |
| **419** | 8 | 30,979 | -3 | 0.007 |
| **411** | 5.3 | 31,862 | 3.2 | 0.008 |
| **409** | 7.5 | 32,180 | -7.2 | 0.006 |
| **402** | 5.7 | 32,692 | 3.1 | 0.023 |
| **400** | 5.6 | 32,736 | 2.9 | 0.045 |
| **378** | 5.7 | 34,264 | -2.6 | 0.022 |
| **369** | 7.7 | 35,253 | -3.1 | 0.023 |
| **360** | 5.6 | 35,967 | -2.9 | 0.031 |
| **362** | 6.2 | 36,011 | -2.7 | 0.027 |
| **358** | 5.7 | 36,011 | 7.2 | 0 |
| **356** | 7 | 36,270 | 5.1 | 0.011 |
| **349** | 7 | 37,206 | -2.6 | 0.035 |
| **340** | 6.8 | 37,568 | 11.2 | 0.001 |
| **333** | 7.2 | 38,453 | -2.7 | 0.024 |
| **332** | 6.7 | 38,649 | -2.7 | 0.003 |
| **298** | 7.9 | 41,015 | 2.8 | 0.005 |
| **297** | 7.4 | 41,045 | 2.8 | 0.006 |
| **292** | 5.6 | 41,338 | 3.3 | 0.011 |
| **290** | 7.6 | 41,527 | -3.8 | 0.013 |
| **283** | 5.6 | 42,081 | 3.2 | 0.006 |
| **279** | 8 | 42,290 | -3.4 | 0.178 |
| **229** | 6.3 | 49,342 | -2.9 | 0.018 |
| **214** | 7.2 | 52,154 | 3.8 | 0.042 |
| **184** | 5.2 | 57,852 | 4.4 | 0.025 |
| **173** | 5.6 | 58,751 | -4 | 0.19 |
| **147** | 7.5 | 63,573 | -9.6 | 0.004 |
| **69** | 8.1 | 82,559 | 3.9 | 0.03 |
| **68** | 8 | 82,792 | 3.4 | 0.023 |
| **44** | 7.4 | 88,830 | -2.9 | 0.011 |
| **30** | 7.5 | 93,453 | -3.7 | 0.268 |
| **26** | 7.9 | 99,201 | 2.7 | 0.003 |
| **22** | 7.3 | 104,801 | -3 | 0.038 |
| **13** | 7.3 | 131,601 | 3.1 | 0.02 |
| **12** | 7.2 | 132,401 | 2.6 | 0.015 |
