# Supplementary material for: Characterization of Antibacterial Activities of Eastern Subterranean Termite, Reticulitermes flavipes, against Human Pathogens
Source: PLoS One. 2016 Sep 9;11(9):e0162249. doi: 10.1371/journal.pone.0162249 (PMC5017719; doi:10.1371/journal.pone.0162249)
Supplement: S2 Table — The pI and the MW of termite hemolymph proteins are based on the Kendrick Labs’ analysis of the two-dimensional gels. (DOCX) [file pone.0162249.s005.docx]

**S2 Table. Differentially expressed hemolymph proteins in MRSA-challenged termites with at least 2.5-fold change.**

| **Spot #** | **pI** | **MW** | **MRSA vs Naïve Difference** | **T-test of MRSA vs Naïve** |
| --- | --- | --- | --- | --- |
|  |  | **(Da)** |  |  |
| **486** | 6.6 | 18,674 | 7.7 | 0.007 |
| **461** | 6.6 | 25,506 | 2.6 | 0.019 |
| **428** | 6 | 26,359 | 3 | 0.01 |
| **450** | 5.7 | 27,591 | 2.5 | 0.013 |
| **449** | 5.9 | 28,081 | -3.4 | 0.004 |
| **440** | 7.1 | 29,320 | 3 | 0.017 |
| **422** | 7.8 | 30,857 | -7.4 | 0.006 |
| **419** | 8 | 30,979 | -6.5 | 0.007 |
| **403** | 8 | 32,692 | 6.7 | 0.008 |
| **381** | 7.5 | 34,163 | 3.6 | 0.043 |
| **319** | 6.9 | 39,471 | 2.5 | 0.014 |
| **315** | 6.8 | 39,774 | -6.2 | 0.025 |
| **308** | 7.6 | 40,162 | -2.8 | 0.007 |
| **298** | 7.9 | 41,015 | -2.8 | 0.043 |
| **283** | 5.6 | 42,081 | -4.2 | 0.011 |
| **254** | 7.2 | 44,637 | 6.3 | 0.035 |
| **249** | 6.4 | 46,320 | -2.7 | 0.024 |
| **243** | 7 | 47,295 | -3.1 | 0.014 |
| **237** | 5.9 | 48,296 | -2.6 | 0.002 |
| **235** | 5.8 | 48,521 | -2.5 | 0.001 |
| **222** | 7.3 | 51,064 | 4 | 0.005 |
| **201** | 7.4 | 54,533 | -8.2 | 0.031 |
| **193** | 7.8 | 55,951 | -4 | 0.003 |
| **194** | 5.9 | 56,053 | -10.4 | 0.001 |
| **192** | 7.7 | 56,066 | -2.8 | 0.008 |
| **189** | 8 | 57,477 | -6.2 | 0.007 |
| **187** | 5.9 | 57,515 | -4.2 | 0.002 |
| **174** | 5.5 | 58,639 | 2.7 | 0.019 |
| **163** | 7.4 | 60,547 | -2.7 | 0.016 |
| **162** | 7.5 | 60,683 | -4.1 | 0.038 |
| **165** | 5.9 | 61,318 | -9.4 | 0.001 |
| **155** | 5.8 | 62,372 | -4.6 | 0.001 |
| **154** | 5.7 | 62,636 | -2.6 | 0.014 |
| **142** | 7.5 | 63,946 | -7.5 | 0.013 |
| **145** | 7.8 | 64,758 | -4.6 | 0.01 |
| **131** | 8.1 | 67,007 | -2.8 | 0.002 |
| **113** | 7.1 | 69,385 | -2.8 | 0.001 |
| **108** | 6.5 | 70,937 | -2.5 | 0.036 |
| **105** | 6.9 | 71,205 | -4.3 | 0.026 |
| **107** | 5.5 | 71,465 | -2.7 | 0.04 |
| **104** | 7 | 71,473 | -4.8 | 0.036 |
| **101** | 6.3 | 71,875 | -3.6 | 0 |
| **94** | 6.4 | 72,947 | -3.5 | 0.005 |
| **82** | 5.6 | 76,473 | -3.9 | 0.015 |
| **81** | 5.5 | 76,605 | -5.7 | 0.001 |
| **80** | 5.7 | 76,736 | -4.1 | 0.001 |
| **79** | 5.8 | 77,264 | -4.9 | 0.01 |
| **76** | 6.5 | 79,378 | -2.6 | 0.012 |
| **75** | 6.4 | 79,512 | -3.8 | 0.004 |
| **74** | 6.3 | 79,780 | -4.5 | 0.033 |
| **70** | 6.8 | 81,924 | -3.7 | 0.03 |
| **67** | 7.8 | 82,792 | -9.2 | 0.03 |
| **62** | 6.6 | 84,068 | -4.4 | 0 |
| **61** | 6.5 | 84,470 | -4.4 | 0.007 |
| **59** | 6.8 | 84,872 | -6.6 | 0.005 |
| **58** | 6.7 | 85,274 | -5.5 | 0.014 |
| **57** | 7.3 | 85,295 | -3.6 | 0.013 |
| **56** | 7.8 | 85,820 | -5.3 | 0.011 |
| **55** | 7.7 | 85,820 | -6.6 | 0.015 |
| **51** | 7.6 | 87,794 | -13.4 | 0.003 |
| **47** | 7.3 | 88,558 | -2.6 | 0.026 |
| **22** | 7.3 | 104,801 | -6.5 | 0.04 |
| **17** | 7.2 | 114,401 | -3 | 0.049 |
| **7** | 7.3 | 169,600 | -3 | 0.042 |
| **164** | Nd | Nd | -2.5 | 0.041 |
